# Potent anti-inflammatory and antinociceptive activity of the endothelin receptor antagonist bosentan in monoarthritic mice

**DOI:** 10.1186/ar3372

**Published:** 2011-06-20

**Authors:** Anne-Katja Imhof, Laura Glück, Mieczyslaw Gajda, Rolf Bräuer, Hans-Georg Schaible, Stefan Schulz

**Affiliations:** 1 Institute of Pharmacology and Toxicology, University Hospital, Friedrich Schiller University, Drackendorfer Str. 1, 07747 Jena, Germany; 2 Institute of Pathology, University Hospital, Friedrich Schiller University, Ziegelmühlenweg 1 07743 Jena, Germany; 3 Institute of Physiology I, University Hospital, Friedrich Schiller University, Teichgraben 8, 07743 Jena, Germany

## Abstract

**Introduction:**

Endothelins are involved in tissue inflammation, pain, edema and cell migration. Our genome-wide microarray analysis revealed that endothelin-1 (ET-1) and endothelin-2 (ET-2) showed a marked up-regulation in dorsal root ganglia during the acute phase of arthritis. We therefore examined the effects of endothelin receptor antagonists on the development of arthritis and inflammatory pain in monoarthritic mice.

**Methods:**

Gene expression was examined in lumbar dorsal root ganglia two days after induction of antigen-induced arthritis (AIA) using mRNA microarray analysis. Effects of drug treatment were determined by repeated assessment of joint swelling, pain-related behavior, and histopathological manifestations during AIA.

**Results:**

Daily oral administration of the mixed ET_A _and ET_B _endothelin receptor antagonist bosentan significantly attenuated knee joint swelling and inflammation to an extent that was comparable to dexamethasone. In addition, bosentan reduced inflammatory mechanical hyperalgesia. Chronic bosentan administration also inhibited joint swelling and protected against inflammation and joint destruction during AIA flare-up reactions. In contrast, the ET_A_-selective antagonist ambrisentan failed to promote any detectable antiinflammatory or antinociceptive activity.

**Conclusions:**

Thus, the present study reveals a pivotal role for the endothelin system in the development of arthritis and arthritic pain. We show that endothelin receptor antagonists can effectively control inflammation, pain and joint destruction during the course of arthritis. Our findings suggest that the antiinflammatory and antinociceptive effects of bosentan are predominantly mediated via the ET_B _receptor.

## Introduction

Rheumatoid arthritis (RA) is a systemic disorder of unknown etiology and is characterized by chronic inflammation and proliferation of the synovial membrane, angiogenesis, and dysregulation of immune responses, which lead to progressive destruction of arthritic joints. A major symptom of RA is chronic recurrent pain, which results from the activation and sensitization of primary afferent nociceptors [1]. After sensitization, nociceptive neurons respond more strongly to mechanical or thermal stimulation. This process is triggered by a number of inflammatory mediators, only some of which (including IL-6, tumor necrosis factor-alpha, bradykinins, and prostaglandins) have been studied in detail [1].

Antigen-induced arthritis (AIA) is a well-established model of experimental arthritis in rodents and shows many similarities to human RA [2,3]. Whereas granulocyte infiltration and edema formation occur during the acute phase of AIA, the chronic phase is characterized by synovitis with infiltration of mononuclear cells into the synovial tissue, angiogenesis, pannus formation, and cartilage and bone erosion. In addition, flare-up reactions can be triggered in a timely manner in this model. We have examined gene expression changes in dorsal root ganglia (DRGs) during the acute phase of AIA. This approach led to the identification of a large number of AIA-regulated genes. Among the genes, which showed a marked upregulation, were several members of the endothelin system, including ET-1, ET-2, and ET_A_.

The endothelin system consists of three peptide ligands (ET-1, ET-2, and ET-3), which bind to two distinct G protein-coupled receptors designated ET_A _and ET_B _[4]. Whereas ET-1 and ET-2 can bind to ET_A _and ET_B_, ET-3 selectively activates ET_B _receptors [4]. ET_A _receptors have been found on small-diameter DRG neurons [5,6]. Activation of these neurons by ET-1 elicits increased excitability by a rise in intracellular Ca^2+ ^and activation of voltage-gated Na^+ ^channels [7]. ET_B _receptors are expressed mainly in DRG satellite cells and Schwann cells [5]. It is thought that ET_B _receptors on these cells can stimulate prostaglandin E_2 _synthesis and release [8,9]. This study was designed to test our hypothesis that the endothelin system could represent a potential target for therapeutic intervention in RA. We therefore examined the effects of endothelin receptor antagonists on the inflammation and inflammatory pain during the course of murine antigen-induced arthritis.

## Materials and methods

### Animals

Experiments were performed on 86 adult female C57BL/6J mice (age range of 12 to 16 weeks and body weight of 20 to 30 g). Animals were housed in a climate-controlled room on a 12-hour light/dark cycle with water and standard rodent chow available *ad libitum*. Ethical approval was obtained before the experiments. All experiments were approved by the Thuringian state authorities and complied with European Community regulations (86/609/EEC) for the care and use of laboratory animals.

### Antigen-induced arthritis

Animals were immunized by subcutaneous injection of 100 μg of methylated BSA (mBSA) (Sigma-Aldrich, Seelze, Germany) dissolved in 50 μL of phosphate-buffered saline (PBS) and emulsified in 50 μL of complete Freund's adjuvant (CFA) (Sigma-Aldrich) 21 and 14 days before induction of AIA. CFA was supplemented with 2 mg/mL heat-killed *Mycobacterium tuberculosis *strain H37RA (Difco, Heidelberg, Germany). In parallel to immunizations, 5 × 10^8 ^heat-inactivated *Bordetella pertussis *germs (Chiron-Behring, Marburg, Germany) were administered intraperitoneally. On day 0, mice were briefly anesthetized with 2.5% isoflurane, and arthritis was induced by injecting 100 μg of sterile mBSA dissolved in 20 μL of PBS into the right knee joint cavity, leading to the development of severe acute synovitis associated with subsequent cartilage and bone erosion in the arthritic joint. Flare-up reactions were provoked by injecting 100 μg of mBSA dissolved in 20 μL of PBS on days 21 and 35 of AIA into the right knee joint cavity.

### mRNA microarray analysis

For microarray analysis, mice in the AIA group (*n *= 3) were immunized with mBSA and AIA was induced in the right knee joint. Mice in the control group (*n *= 3) were immunized with mBSA but received an injection of saline into the right knee joint. On day 2 of AIA, mice were killed by cervical dislocation, and lumbar DRGs (L_3_-L_5_; ipsi- and contralateral) were dissected and immediately frozen in liquid nitrogen. Successful induction of AIA was verified by measurement of joint swelling and histopathological examination. Total RNA was extracted by using RNeasy (Qiagen, Hilden, Germany) and hybridized onto an Illumina MouseWG-6 version 1.1 Expression BeadChip (Illumina, Inc., San Diego, CA, USA) at SIRSLab (Jena, Germany). Fold change of expression was defined as (AIA left - control left)/(AIA right - control right), which includes a normalization to controls. All bead types with a *P *value of less than 0.01 and fold change of at least 5.0 and not more than -5.0 were selected for further examination by using Ingenuity Pathways Analysis Software (Ingenuity Systems, Inc., Redwood City, CA, USA). Microarray data have been deposited in a public database [10].

### Treatment protocol and drugs

Drug treatment was similar to that reported in previous studies [11,12]. Briefly, mice were allocated to the following groups of 10 animals each under randomized conditions: 0.9% saline *per os *(p.o.), bosentan 100 mg/kg p.o., and ambrisentan 10 mg/kg p.o. Bosentan and ambrisentan were dissolved in saline and administered orally in a volume of 10 mL/kg body weight. Bosentan (RO470203) was obtained from Actelion (Basel, Switzerland). Ambrisentan (LU208075) was provided by Gilead Sciences (Foster City, CA, USA). Treatment started 2 hours before induction of AIA and was continued every 24 hours for the indicated time periods (3, 21, or 42 days). An additional group received 0.6 mg/kg dexamethasone palmitate (Merckle, Ulm, Germany) by intraperitoneal injection. Dexamethasone treatment was carried out for 5 days followed by a 2-day pause starting 12 hours before AIA.

### Pain-related behavior and clinical inflammation measurement

At two time points before AIA induction (baseline) and on days 1, 3, 7, 14, and 21 of AIA, secondary mechanical hyperalgesia was determined on ipsi- and contralateral hindpaws by using a dynamic plantar aesthesiometer (Ugo Basile, Comerio, Italy). Animals were placed on a mesh floor and allowed to acclimate to the testing device. Then an automated blunt filament was directed to the plantar surface of the paw, and pressure was increased until the animal withdraws its limb. The weight force needed to elicit this response was read out in grams. In this study, 10 g were defined as cutoff. Measurements were performed in triplicate, and means were taken as mechanical hyperalgesic thresholds. Secondary thermal hyperalgesia was assessed at hindpaws with an algesiometer (Ugo Basile) as described [2,13]. After acclimation of the animals to the testing device, three consecutive radiant heat stimuli were applied to the hindpaws with intervals of at least 1 minute between stimuli. Mean latencies were calculated and used as a measure of withdrawal threshold to heat. Stimuli were applied for a maximum of 10 seconds to prevent tissue damage. Swelling was assessed on days 0 to 5, 7, 14, and 21 of AIA by measuring the mediolateral diameter of each knee by means of an Oditest caliper (Kroeplin, Schlüchtern, Germany). For each animal and test day, swelling was calculated by subtracting the diameter of the noninflamed knee from that of the inflamed knee to account for anatomical knee joint differences between animals.

### Histopathological grading of joint inflammation and destruction

Tissues were obtained immediately after the final testing. Both knee joints were removed, skinned, fixed in 4% formalin, decalcified with 15% EDTA (ethylenediaminetetraacetic acid) for 5 days or in 7% AlCl_3 _in 2.1% HCl and 6% formic acid for 48 hours, embedded in paraffin, cut into 3-μm thick frontal sections, and stained with hematoxylin-eosin for microscopic examination. Four sections from different levels of the knee joint were examined by an independent observer who was blinded to the treatments and were evaluated according to a histological scoring system ranging from 0 to 3 (0 = no, 1 = mild, 2 = moderate, and 3 = severe alterations). The amount of fibrin exudation and the relative number and density of granulocytes in synovial membrane and joint space allowed grading of the acute inflammatory reaction, and the relative number and density of infiltrating mononuclear leukocytes in the synovial membrane, the degree of synovial hyperplasia, and the extent of infiltration and fibrosis in the periarticular structures allowed grading of chronic inflammation. The extent of damage of the cartilage surface and bone structures was also evaluated on a scale of 0 to 3, where 0 = no damage, 1 = mild destruction, 2 = moderate destruction, and 3 = severe destruction of cartilage and bone (extensive area of chondrocyte death and cartilage destruction and deep invasive bone erosions) [14].

### Statistical analyses

**Figure 1:**
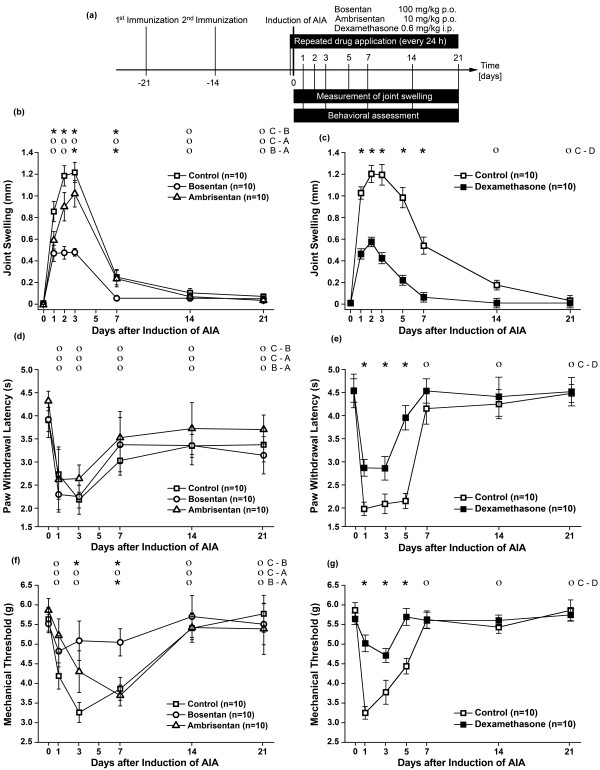
**Effects of bosentan and ambrisentan on antigen-induced arthritis (AIA)**. (a) Schematic drawing of experimental setup. Animals were immunized 21 and 14 days before induction of AIA. Mice received repeated oral applications of 100 mg/kg bosentan, 10 mg/kg ambrisentan, or saline (Control) every 24 hours beginning 2 hours before induction of AIA. Dexamethasone was given intraperitoneally (i.p.) at a dose of 0.6 mg/kg for 5 days beginning 12 hours before induction of AIA. Joint swelling and pain-related behavior were assessed as indicated. All animals were tested twice during the immunization procedure to obtain baseline values depicted as day 0. **(b) **Inhibition of knee joint swelling by bosentan but not by ambrisentan. Knee joint swelling as an indicator of inflammation was assessed by measuring the mediolateral diameter of each knee. **(c) **Inhibition of knee joint swelling by dexamethasone. **(d) **Lack of inhibition of thermal hyperalgesia by bosentan or ambrisentan. Thermal hyperalgesia was determined with an algesiometer and calculated as reduced withdrawal threshold to heat. **(e) **Inhibition of thermal hyperalgesia by dexamethasone. **(f) **Inhibition of mechanical hyperalgesia by bosentan but not by ambrisentan. Mechanical hyperalgesia was determined on ipsi- and contralateral hindpaws by using a dynamic plantar aesthesiometer. The weight force needed to elicit a response was read out in grams. **(g) **Inhibition of mechanical hyperalgesia by dexamethasone. Values in (b-e) are means ± standard error of the mean. The results from two-way analysis of variance followed by the Bonferroni *post hoc *test are shown (*P < 0.05; ο, not significant). p.o., *per os *(by mouth).

**Figure 2:**
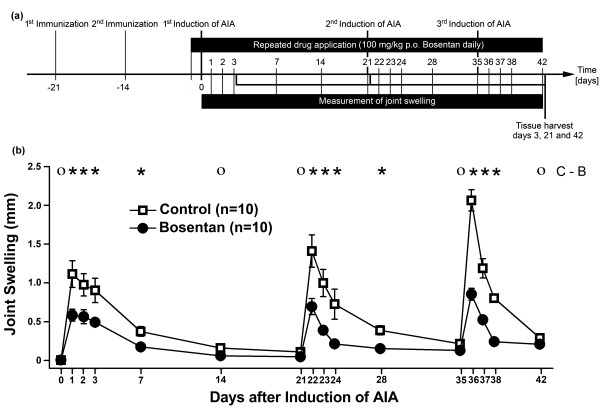
**Effect of bosentan on antigen-induced arthritis (AIA) flare-up reactions**. (a) Schematic drawing of experimental setup. Animals were immunized 21 and 14 days before induction of AIA. Mice received repeated oral applications of either 100 mg/kg bosentan or saline (Control) every 24 hours for 42 days beginning 2 hours before the initial induction of AIA. AIA flare-up reactions were provoked on days 21 and 35. Joint swelling was assessed as indicated. All animals were tested twice during the immunization procedure to obtain baseline values depicted as day 0. After 3, 21, or 42 days, mice were killed, and affected knee joints were prepared for histological scoring. **(b) **Inhibition of knee joint swelling by bosentan during AIA flare-up reactions. Knee joint swelling as an indicator of inflammation was assessed by measuring the mediolateral diameter of each knee. Values in (b) are means ± standard error of the mean. The results from two-way analysis of variance followed by the Bonferroni *post hoc *test are shown (*P < 0.05; ο, not significant). p.o., *per os *(by mouth).

**Figure 3:**
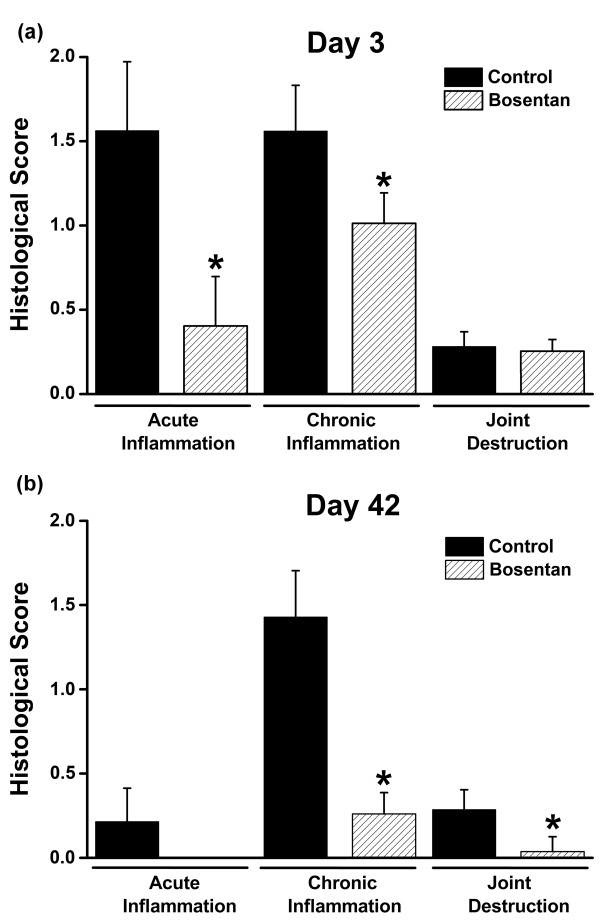
**Effect of bosentan on histopathological manifestations of antigen-induced arthritis (AIA)**. (a) Mice were killed 3 days after induction of AIA. **(b) **Mice were killed at day 42 after repeated induction of AIA. Affected knee joints were prepared for histological scoring. Four sections per knee joint were examined by an observer who was blinded to the treatments and were scored according to a three-parameter scoring system as described in Materials and methods. Values are means ± standard error of the mean. The results from two-way analysis of variance followed by the Bonferroni *post hoc *test are shown (*P < 0.05).

For statistical analyses, SPSS for Windows (version 17.0; SPSS Inc., Chicago, IL, USA) was used. First, data were tested for normal distribution by applying the Kolmogorov-Smirnov test. Differences in histopathological scores for acute inflammation, chronic inflammation, and joint destruction as well as joint swelling were analyzed by one-way analyses of variance (ANOVAs) followed by *post hoc t *tests for comparison between different groups. Measures obtained from different time points were compared between groups by using repeated measures ANOVAs with the between-subjects factor 'group' (vehicle, bosentan, and ambrisentan) and the within-subjects factor 'time' (baseline and days 1, 3, 7, 14, and 21 after induction of AIA). *Post hoc t *tests were used to describe differences between groups at different time points when ANOVAs revealed a significant main effect. Significance was accepted for *P *values of less than 0.05. *P *values from *post hoc *tests are displayed in Figures 1, 2, 3 whenever multivariate tests show significant overall effects.

## Results

### Effects of endothelin receptor antagonists on antigen-induced arthritis

We have assessed gene expression changes in lumbar DRGs during the acute phase of AIA by using transcriptional profiling by genome-wide microarray analysis. Intriguingly, three members of the endothelin system - namely ET-1, ET-2, and ET_A _- were also strongly upregulated (Table 1). ET_B _was also detected during array analysis but was not regulated (Table 1). The upregulation of ET-1 and ET-2 was then verified by real-time polymerase chain reaction (data not shown). We therefore evaluated effects of the mixed ET_A _and ET_B _endothelin receptor antagonist bosentan and the ET_A_-selective antagonist ambrisentan on AIA in mice. Mice received daily oral administrations for 21 days beginning 2 hours before induction of AIA. Knee joint swelling and pain-related behavior were assessed repeatedly during the course of AIA (Figure 1a). On days 1 to 5, untreated mice with AIA exhibited pronounced swelling of the injected knee, which slowly subsided until day 21 (Figure 1b). Bosentan strongly inhibited joint swelling during the acute phase of AIA (Figure 1b). In contrast, ambrisentan failed to promote any detectable anti-inflammatory effect (Figure 1b). Under these conditions, the anti-inflammatory activity of bosentan was similar to that observed after administration of dexamethasone (Figure 1c). Untreated mice with AIA also exhibited secondary thermal hyperalgesia, which was detected as decreased withdrawal latency to radiant heat (Figure 1d). Neither bosentan nor ambrisentan significantly increased latencies until paw withdrawal at the inflamed side (Figure 1d). In contrast, repeated application of dexamethasone produced a detectable inhibition of thermal hyperalgesia (Figures 1e). Untreated mice with AIA also exhibited secondary mechanical hyperalgesia, which was detected as decreased withdrawal threshold to mechanical stimuli (Figure 1f). Like mice treated with dexamethasone, bosentan-treated mice showed significantly increased mechanical thresholds at the inflamed side (Figure 1f,1g). These findings indicate that the mixed ET_A _and ET_B _endothelin receptor antagonist bosentan elicits robust anti-inflammatory and antinociceptive responses in monoarthritic mice, whereas the ET_A_-selective antagonist ambrisentan failed to promote any detectable anti-inflammatory or antinociceptive activity.

**Table 1:** Selected genes that are upregulated in dorsal root ganglia two days after induction of antigen-induced arthritis as determined by microarray analysis

Illumina ID	Gene	Synonym	Fold change
scl0011829.2_75-S	*AQP4*^a^	Aquaporin 4	9
scl27591.6.1_80-S	*AREG*^a^	Amphiregulin	10
scl27547.3.1_4-S	*BMP3*	Bone morphogenetic protein 3	8
scl26388.10_270-S	*BTC*	Betacellulin	5
scl026365.2_7-S	*CEACAM1*	Carcinoembryonic antigen-related cell adhesion molecule	13
scl0023844.2_19-S	*CLCA3*	Ca^2+^-activated chloride channel	1,724
scl020312.5_187-S	*CX3CL1*^a^	Chemokine (C-X3-C motif) ligand 1	6
scl48937.1.1_21-S	*CXADR*	Coxackie and adenovirus receptor	15
scl31983.48.1_26-S	*DMBT1*	Deleted in malignant brain tumors	678
scl44852.5.1_6-S	*EDN1*^a^	Endothelin 1	16
scl25019.5.1_161-S	*EDN2*	Endothelin 2	5
scl15480.1.1_277-S	*EDNRA*^a^	Endothelin receptor A	7
scl45193.8_18-S	*EDNRB*	Endothelin receptor B	1
scl0001767.1_56-S	*FAM3B*	Family with sequence similarity 3, member B	10
scl43662.2_474-S	*F2RL1*	F2RL1 coagulation factor II receptor-like 1	28
scl48150.3.1_29-S	*FAM3D*	Family with sequence similarity 3, member D	18
scl47093.2_645-S	*GPR20*	G protein-coupled receptor 20	22
scl53162.3.1_182-S	*GPR120*	G protein-coupled receptor 120	6
scl0232431.4_71-S	*GPRC5A*	G protein-coupled receptor, family C, group 5, member A	62
scl25025.4.1_56-S	*GUCA2A*	Guanylin	802
scl016173.8_28-S	*IL18*^a^	Interleukin 18	6
scl49177.8_486-S	*ILDR1*	Ig-like domain-containing receptor 1	26
scl016612.5_71-S	*KLK1*^a^	Kallikrein 1	113
GI_6754459-S	*KLK1B26*	Kallikrein 1-related peptidase b26	112
scl018050.1_7-S	*KLK1B4*	Kallikrein 1-related peptidase b4	127
scl000139.1_0-S	*KLK1B5*	Kallikrein 1-related peptidase b5	302
scl0016619.1_79-S	*KLK3*	Kallikrein 1-related peptidase b27	121
scl49904.15_203-S	*MEP1A*	Meprin A, alpha	20
scl48741.4.1_176-S	*PLA2G4F*^a^	Phospholipase A2	93
scl39519.5.1_59-S	*PYY*	Peptide YY	72
scl16482.8_0-S	*RAB17*	Member of RAS oncogene family	9
scl24993.3_35-S	*RHBDL2*	Rhomboid, veinlet-like 2	5
scl22946.3.1_72-S	*S100A14*^a^	S100 calcium-binding protein	55
scl32104.13.1_7-S	*SCNN1B*	Na-channel, nonvoltage-gated 1, beta-subunit	7
scl30493.4.19_120-S	*SCT*^a^	Secretin	9
scl20135.8.1_22-S	*SDCBP2*	Syndecan-binding protein (syntenin) 2	41
scl026456.19_173-S	*SEMA4G*	Semaphorin 4G	9
scl0020510.2_224-S	*SLC1A1*	Solute carrier family 1, member 1	5
scl32784.15.1_26-S	*SLC7A9*	Solute carrier family 7, member 9	11
scl00226999.1_58-S	*SLC9A2*	Solute carrier family 9, member 2	31
scl00171286.2_214-S	*SLC12A8*	Solute carrier family 12, member 8	16
scl39885.12.1_61-S	*SLC13A2*	Solute carrier family 13, member 2	47
scl47037.12.1_89-S	*SLC39A4*	Solute carrier family 39, member 4	16
scl41202.6.1_16-S	*SLC46A1*^a^	Solute carrier family 46, member 1	5

Genes were annotated by using Illumina (San Diego, CA, USA) and National Center for Biotechnology Information databases. ^a^Genes previously associated with arthritis or inflammatory pain.

### Effect of bosentan on antigen-induced arthritis flare-up reactions

Given the potent anti-inflammatory and antinociceptive activity of bosentan during a single induction of AIA, we asked whether bosentan could protect against repeated induction of AIA. Mice received oral administration of bosentan every 24 hours for 42 days beginning 2 hours before the initial induction of AIA. AIA flare-up reactions were provoked 21 and 35 days later by injection of mBSA into the knee joint cavity. Knee joint swelling was assessed repeatedly during the course of AIA (Figure 2a). As depicted in Figure 2b, untreated mice responded with a pronounced increase in joint swelling during each AIA flare-up reaction. Bosentan significantly inhibited joint swelling during each of these flare-up reactions (Figure 2b). Weight loss or any other easily detectable unwanted drug effects were not noted during the 42-day treatment period. As shown in Figure 3, bosentan also potently suppressed histopathological manifestations of acute and chronic inflammation detected 3 days after AIA induction as well as inflammation and joint destruction during AIA flare-up reactions.

## Discussion

In an effort to examine gene expression changes during experimental arthritis, we found that three members of the endothelin system - namely ET-1, ET-2, and ET_A _- were markedly upregulated during the acute phase of AIA. This is in line with previous findings showing that patients with RA exhibit increased ET-1 serum levels as well as high ET-1 concentrations in synovial fluid [15-17]. Moreover, it is widely accepted that endothelins induce hypernociception in rodents [18-22]. So far, studies investigating the role of endothelins in the pathophysiology of arthritis are sparse [18,23,24]. It has been shown, however, that local administration of endothelin receptor antagonists reduces edema, neutrophil infiltration, and production of inflammatory mediators [21,25-32].

Given the availability of potent endothelin receptor antagonists, we investigated the effects of systemic administration of the mixed ET_A _and ET_B _endothelin receptor antagonist bosentan and the ET_A_-selective antagonist ambrisentan on pain-related behavior, inflammation, and histopathological manifestations during the course of AIA. We found that daily oral administration of bosentan significantly attenuated knee joint swelling. In contrast, ambrisentan failed to promote any detectable anti-inflammatory activity. These findings indicate that the anti-inflammatory effects of bosentan are mediated predominantly via the ET_B _receptor.

Bosentan selectively inhibited mechanical hyperalgesia but not thermal hyperalgesia. Acute and chronic models of joint inflammation reliably produce mechanical hyperalgesia. In some arthritic models, thermal hyperalgesia can also be observed; however, it is not known to what extent thermal hyperalgesia is important in humans. Interestingly, intradermal injection of ET-1 induces mechanical hyperalgesia in humans, whereas thermal hyperalgesia could not be observed. Moreover, previous findings revealed different contributions of ET_A _and ET_B _receptors to thermal and mechanical hyperalgesia, respectively [2,9,21,25,28,29,31-34]. Whereas ET_A _receptors have been shown to mediate ET-1-induced thermal hyperalgesia, ET_B _receptors have been linked to mechanical hyperalgesia [2,9,21,25,28,29,31-34]. Both ambrisentan and bosentan had no effect on thermal hyperalgesia. In contrast, dexamethasone produced a significant inhibition of thermal hyperalgesia, suggesting that mechanisms in addition to an upregulation of ET-1 or ET-2 may contribute to the development of thermal hyperalgesia in our AIA model. At present, we do not know whether ET_B_-selective antagonists could exert therapeutic effects similar to those of mixed ET_A _and ET_B _receptor antagonists. Nevertheless, daily oral bosentan administration was well tolerated over the 42-day treatment period in our murine AIA model.

To assess gene expression changes in lumbar DRGs during the acute phase of AIA, we used transcriptional profiling by genome-wide microarray analysis. Our results indicate that an acute peripheral inflammation of the knee joint induces robust changes in gene expression patterns in DRGs, suggesting that dynamic adaptations occur in primary sensory neurons in response to peripheral inflammation. However, this approach is based on the isolation of total mRNA from DRGs and, hence, cannot differentiate between mRNAs originating from neurons, glial cells, endothelial cells, or infiltrating leukocytes. Nevertheless, we detected a total of 451 AIA-regulated genes, 436 of which were upregulated (fold change of at least 5) and only 15 of which were downregulated (fold change of not more than -5) in DRGs from the affected side in comparison with the contralateral side and control animals. Table 1 shows a selection of upregulated genes. This selection includes regulatory peptides (for example, secretin, peptide YY, and guanylin) as well as chemokines, receptors, enzymes, and carriers. Several of these genes, including phospholipase A2, kallikrein, IL-18, and CX3CL1, have been associated with arthritis or inflammatory pain.

## Conclusions

We identify the endothelin system as a potential target for therapeutic intervention in RA by mRNA microarray analysis. We clearly show that chronic oral bosentan administration inhibits joint swelling, protects against joint inflammation and destruction, and reduces mechanical hyperalgesia during AIA induction and during AIA flare-up reactions. Thus, our findings on the endothelin system provide proof of concept that global gene expression profiling can lead to the identification of novel therapeutic targets in arthritis.

## Abbreviations

AIA: antigen-induced arthritis; ANOVA: analysis of variance; CFA: complete Freund's adjuvant; DRG: dorsal root ganglion; ET-1: endothelin-1; ET-2: endothelin-2; ET_A_: endothelin receptor A; ET_B_: endothelin receptor B; IL: interleukin; mBSA: methylated bovine serum albumin; PBS: phosphate-buffered saline; p.o.: *per os *(by mouth); RA: rheumatoid arthritis.

## Competing interests

The authors declare that they have no competing interests.

## Authors' contributions

A-KI carried out the experiments and drafted the manuscript. LG carried out the experiments and helped to draft the manuscript. MG carried out the histopathological examination. RB and H-GS participated in the design of the study and helped to draft the manuscript. SS conceived the study, participated in its design and coordination, and helped to draft the manuscript. All authors read and approved the final manuscript.
